# Continuous exposure to doxorubicin induces stem cell-like characteristics and plasticity in MDA-MB-231 breast cancer cells identified with the SORE6 reporter

**DOI:** 10.1007/s00280-024-04701-4

**Published:** 2024-08-24

**Authors:** Nohemí Salinas-Jazmín, María Adriana Medina-Mondragón, Jeannie Jiménez-López, Sandra Lucia Guerrero-Rodríguez, Patricia Cuautle-Rodríguez, Marco Antonio Velasco-Velázquez

**Affiliations:** 1 Departamento de Farmacología, Facultad de Medicina, Universidad Nacional Autónoma de México (UNAM), 04510 Ciudad de Mexico, México; 2 Posgrado en Ciencias Bioquímicas, UNAM, 04510 Ciudad de Mexico, México

**Keywords:** Breast cancer, Doxorubicin, CSC-like, Plasticity, Drug-resistance

## Abstract

**Purpose:**

Cancer stem cells (CSCs) account for recurrence and resistance to breast cancer drugs, rendering them a cause of mortality and therapeutic failure. In this study, we examined the effects of exposure to low concentrations of doxorubicin (Dox) on CSCs and non-CSCs from TNBC.

**Methods:**

The effects of Dox were studied using the SORE6 reporter system. We examined the enrichment of the CSCs population, as well as the proliferation, and death of the reporter-positive fraction (GFP + cells) by flow cytometry. The resistant and stemness phenotypes were analyzed by viability and mammosphere formation assay, respectively. We identified differentially expressed and coregulated genes by RNA-seq analysis, and the correlation between gene expression and clinical outcome was evaluated by Kaplan-Mayer analysis using public databases.

**Results:**

In MDAMB231 and Hs578t cells, we identified enriched subsets in the CSCs population after continuous exposure to low concentrations of Dox. Cells from these enriched cultures showed resistance to toxic concentrations of Dox and increased efficiency of mammosphere formation. In purified GFP + or GFP- cells, Dox increased the mammosphere-forming efficiency, promoted phenotypic switches in non-CSCs populations to a CSC-like state, reduced proliferation, and induced differential gene expression. We identified several biological processes and molecular functions that partially explain the development of doxorubicin-resistant cells and cellular plasticity. Among the genes that were regulated by Dox exposure, the expression of ITGB1, SNAI1, NOTCH4, STAT5B, RAPGEF3, LAMA2, and GNAI1 was significantly associated with poor survival, the stemness phenotype, and chemoresistance.

**Conclusion:**

The generation of chemoresistant cells that have characteristics of CSCs, after exposure to low concentrations of Dox, involves the differential expression of genes that have a clinical impact.

**Supplementary Information:**

The online version contains supplementary material available at 10.1007/s00280-024-04701-4.

## Introduction

Cancer is a fatal disease, with 2.3 million of the total new cases worldwide, of which breast cancer has the highest incidence and mortality in women [1]. The clinical course and prognosis of breast cancer and the responsiveness to treatment depend on its molecular characteristics or classification. Its immunohistochemical classification is based on hormone receptor (HR) expression (estrogen receptor and progesterone receptor), as well as amplification of the human epidermal growth factor receptor ERBB2/HER2-, resulting in the HR-positive (luminal A or B), HER2-positive, and triple-negative (TNBC) subtypes [2, 3].

The standard first-line treatments for TNBC—entailing cytotoxic chemotherapy, such as doxorubicin (Dox), an anthracycline—have a wide range of cytotoxic side effects that are mediated through several mechanisms, including DNA intercalation, topoisomerase II inhibition, and free radical formation [4–7]. However, their therapeutic efficacy is severely limited by the rapid development of drug resistance in cancer cells shortly after the initiation of treatment, resulting in tumor metastasis and recurrence, the main cause of over 90% of cancer-associated deaths [8, 9]. Several reports suggest that the development of resistance to doxorubicin involves cancer cells that acquire multidrug-resistant phenotypes [10], populations (CD44^+^/CD24 ^−/low^) of breast cancer stem cells (CSCs) [11, 12], and initiation of the epithelial-mesenchymal transition (EMT) [13].

CSCs represent a small population within the various types of cancer. In addition to the hallmark alterations of cancer cells, CSCs have the capacity to self-renew and generate a pool of transit-amplifying cells, ultimately contributing to the production of the tumor bulk. Further, CSCs can mediate metastasis and the resistance to cytotoxic treatments, leading to minimal residual disease and cancer relapse in several cancer types, including TNBC [11, 14, 15]. They are considered the root cause of tumor metastasis and recurrence and are characterized by active DNA repair, high expression of ATP-binding cassette (ABC) transporters, anti-apoptosis properties, and a plastic cell phenotype [16].

Several studies have determined some of the mechanisms by which chemotherapy promotes phenotypic switches in non-CSC populations to a CSCs state in cancer cells, correlating them with clinical drug resistance [17–19] and a poor prognosis [20]. However, other signaling pathways that are involved in CSC enrichment in solid cancers remain to be identified. Recent studies have reported that dormant CSCs are generated in response to therapies and can cause recurrence. Tumor dormancy allows cancer cells to survive, altering gene expression, slowing proliferation, and decreasing metabolism, thereby reducing the effects of anticancer drugs [21].

In this study, we established doxorubicin-resistant MDA-MB-231 and Hs578t breast cancer cell lines; these lines have been transduced with the SORE6 reporter system, as described [22]. We assessed the expansion of doxorubicin-resistant CSC-like cells by measuring GFP expression. In addition, the phenotype of doxorubicin-resistant cells was characterized by viability assay and mammosphere formation assay. Doxorubicin increased the mammosphere-forming efficiency from purified cell cultures (GFP- or GFP +), promoted phenotypic switches in non-CSC populations to a CSC-like state, reduced proliferation, and induced differential gene expression. We found that ITGB1, SNAI1, NOTCH4, STAT5B, RAPGEF3, LAMA2, and GNAI participate in several biological processes and molecular functions that partially explain the development of doxorubicin-resistant cells and cellular plasticity.

## Methods

### Cell culture and drug

We obtained MDA-MB-231 (HTB-26) and Hs578t (HTB-126) TNBC cells from American Type Culture Collection (ATCC). Only cells below Passage 20 were used for our experiments. MDA-MB-231 cells were cultured routinely in Leibovitz's L-15 with 10% fetal bovine serum (FBS, F3135 Sigma-Aldrich) at 37 °C in a humidified atmosphere without CO_2_. Hs578t and HEK293T (CRL-3216) cells were grown routinely in Dulbecco’s Modified Eagle Medium (DMEM), supplemented with 10% FBS, at 37 °C in an atmosphere with 5% CO_2_. Doxorubicin (D1515 Sigma-Aldrich) was prepared in Milli Q water and stored at -70 °C with protection from light.

### Identification and isolation of GFP + cells (CSC-like)

The identification and quantification of GFP + cell populations (CSC-like) were performed in sublines that were generated by lentiviral transduction of a reporter system that contained 6 concatenated repeats of SOX2/OCT4 response elements, which drive the expression of destabilized green fluorescent protein (SORE6-GFP). A minimal cytomegalovirus promoter, (mCMVp)-GFP, was used to generate control cell lines. Both reporter constructs were kindly provided by Dr. L.M. Wakefield (National Cancer Institute, Bethesda, MD, USA) [22]. Lentiviral particles were generated SORE6-GFP and mCMVp-GFP, as previously described. HEK293T cells were transfected with the psPAX2 packaging plasmid and the pMD2.G envelope plasmid using Lipofectamine 3000 (L3000-001, Invitrogen)[23]. For transduction with lentiviral particles, MDA-MB-231 or Hs578t cells were exposed to lentiviral supernatants diluted 1:1 in fresh medium for 24 h with 8 μg/ml Polybrene (28728–55-4, Santa Cruz). Transduced cells were positively selected through a sequential treatment with puromycin 0.5 μg/mL (P4512, Sigma-Aldrich) for additional 72 h and GFP-based cell sorting (FACS Aria II Cell Sorter).

MDA-MB-231 SORE6 cells were used to isolate GFP + and GFP- populations by flow cytometry. For purification of GFP + and GFP- populations after 19–22 weeks of exposure to doxorubicin, cultured cells were sorted based on the expression of GFP + using gates that were defined by the control mCMVp-GFP. Cell sorting was performed on a FACSAria, and cells were used immediately for the mammospheres assay, CellTrace violet staining or RNA extraction. The purity of cells was > 95% for each population.

### MTT cell viability assay

The effects of doxorubicin on viability were determined in cells that were in the exponential growth phase by MTT (3-(4,5-Dimethylthiazol-2-yl)-2,5-Diphenyltetrazolium Bromide] assay. Cells were seeded in 96-well plates at a density of 1 × 10^4^ cells/well. Cells were treated with and without doxorubicin as indicated (0–10 μM for MDA-MB-231 or 0-1000 nM for Hs578t). After 72 h, 20 μL MTT solution (5 mg/mL) was added for 3 h at 37 °C. The resulting formazan crystals were dissolved in 100 μL of DMSO. The amount of reduced tetrazolium salt was measured spectrophotometrically at 570 nm (Epoch, Biotek). By nonlinear regression of 4 parameters, we obtained the IC50 and IC75 values (GraphPad Prism v6.0).

### Establishment of doxorubicin-resistant MDA-MB-231-SORE6 cells

Resistant cells were established by continuous and gradual exposure at doxorubicin at increasing concentrations (1–10 nM). The concentration range of doxorubicin was determined, according to our dose–response curve (Supplementary Fig. 1). A passage was performed when the culture was 70% to 80% confluent; each passage was considered to be 5–7 days. Acquisition of resistance occurred at approximately 19–22 weeks. The cells were considered to be resistant at peak viability compared with control cells at IC 50 and IC75 (evaluated by MTT cell viability assay***)***. The cultures (control and Dox) were maintained under identical conditions and for the same period to allow for appropriate comparison between the 2 conditions.

### Quantification of apoptosis by flow cytometry

To evaluate the intrinsic resistance of MDA-MB-231-SORE6 TNBC cells, we exposed them to doxorubicin (IC50) and evaluated their viability by flow cytometry using Annexin V-Alexa Fluor 647 (A23204, Invitrogen)/7AAD (A9400, Sigma). Briefly, cells were seeded in 24-well plates at a density of 50 × 10^4^ cells/well. Cells were treated with and without doxorubicin (0.43 μM) for 72 h. After incubation, the cells were collected in PBS (pH 7.4) and centrifuged for 5 min at 12,000 rpm. The cells were incubated in 300 μl annexin-binding buffer at room temperature for 5 min and double-stained with Annexin V-Alexa Fluor 647 and 7AAD for 15 min and 20 min, respectively. The stained cells were acquired on a Attune NxT cytometer (Applied Biosystem, Attune NxT), and the data were analyzed in FlowJo, V.10.0.

### Mammosphere formation assay

Mammosphere formation assay was performed as reported [24]. Briefly, purified and unpurified cells were plated at low density (100 viable cells per well) on a 96-well ultra-low attachment plate (Corning Costar) with MammoCult medium and growth factors (05620, StemCell Technologies). The number of mammospheres with diameter > 60 μm was quantified on Day 7 by taking micrographs (Eclipse Ti-U microscopy, Nikon) and analyzed in NIS-Elements Imaging Software 4.13. The results are expressed as mammospheres-forming efficiency (MFE), which was calculated with the following equation: $MFE= \frac{N\text{umber of mammospheres per well}}{\text{Number of cells seeded per well}} \times 100$.

### CellTrace violet staining

CellTrace Violet (C34571, Molecular Probes) staining was carried out per the manufacturer's instructions. In brief, isolated cells (GFP + or GFP- populations) were stained with 2.5 μM CellTrace Violet in PBS (5 min, room temperature, in the dark) with occasional stirring. Staining was quenched with the addition of 5 volumes of PBS that contained 10% FBS; the cells were then centrifuged (5 min, 1200 rpm), resuspended in complete L-15 medium, and cultured immediately. Data are expressed as Fold dye dilution of CellTrace (FDD) and was calculated with next equation:  $FDD= \frac{\text{median fluorescence intensity }(t0)}{\text{median fluorescence intensity }(t72 h)}$.

### RNA-seq and analysis of gene expression

Total RNA was extracted from the various cells using the RNeasy Kit (74004 Qiagen). RNA quality was assessed (Agilent Bioanalyzer); all RIN values were greater than 7.0. RNA libraries were generated (KAPA RNA) for RNA-sequencing by 50-bp paired-end sequencing in replicate (Illumina HiSeq 2500). In total, 10^7^ reads were mapped using UCSC hg19, and we followed a workflow to detect differentially expressed (DE) genes and gene ontologies from raw RNA-Seq data using Galaxy. DESeq2 was then used to normalize the read counts and extract DE genes. Heatmap2 and Volcano Plot were used to visualize DE genes in GSEA v2.2.0 using the MSigDB database v5.1. Functional enrichment analysis of the DE genes was performed using g:Profiler version e111_eg58_p18_30541362 (database updated in 2024).

The heat maps and correlations between *genes* in the same patient cohort were verified and analyzed by data mining in TCGA-BRCA using the UCSC Xena browser; also, survival curves were generated (http://xena.ucsc.edu/). Overall survival (OS) was defined as the time from the first diagnosis of primary breast cancer to death from any cause. All results are displayed as P values by log-rank test.

## RT-qPCR

Fifty nanogram of total RNA obtained from isolated populations of GFP + and GFP- were reverse transcribed by using SuperScript II Reverse Transcriptase (18064014, Invitrogen). The cDNAs were diluted 10 times before amplification with iTaq Universal SYBR Green Supermix (1725121, Bio-Rad) using MyGo Pro thermo-cycle. All the samples were normalized to GAPDH housekeeping gene (GTCTCCTCTGACTTCAACAGCG forward; ACCACCCTGTTGCTGTAGCCAA reverse, SIGMA) and the relative expression levels of the genes of interest (SOX2: GCTACAGCATGATGCAGGACCA forward, TCTGCGAGCTGGTCATGGAGTT reverse; OCT4: CCTGAAGCAGAAGAGGATCACC forward, AAAGCGGCAGATGGTCGTTTGG reverse, IDT Integrated DNA Technologies) were calculated using the Efficiency-corrected relative quantification method. The thermal cycling program started with a step of 30 s at 95 °C, followed by 40 cycles (15 s at 95 °C and 60 s at 60 °C). The gene expression levels were compared with student´s test and data are presented as the mean ± standard error of the mean (SEM).

### Statistical analysis

Half-maximal inhibitory concentration (IC_50_ and IC_75_) values were calculated by non-linear regression. Statistical significance was determined by one-way ANOVA or Tukey's or student's test. P values ≤ 0.05 are reported. GraphPad Prism (v6.0) was used to perform these analyses.

## Results

### Doxorubicin-induced enrichment of GFP + cells (CSC-like) in TNBC cells

Cancer stem cells (CSCs) are highly associated with the development of drug-resistant cancer cells, and TNBC is known for its high CSC population [22, 25, 26]. In this study, we used a reporter system (SORE6) [22] that allows the real-time monitoring of such properties as phenotypic plasticity and the response to therapeutic agents in CSC-like (GFP +) cells.

**Fig. 1:**
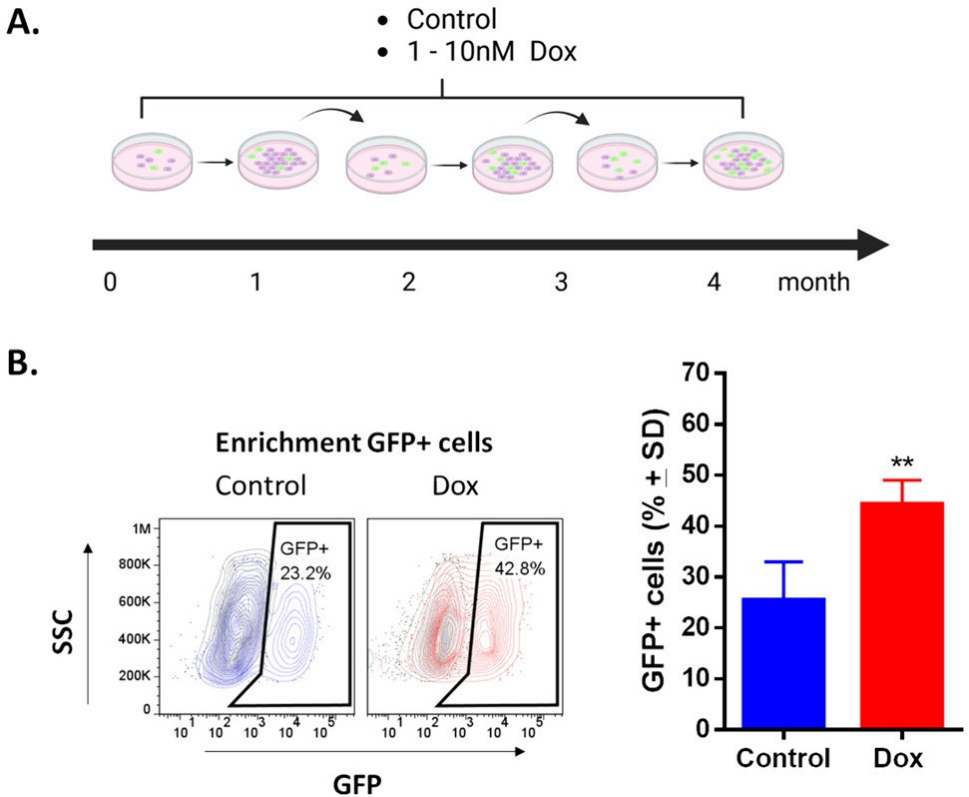
Exposure to low concentrations of doxorubicin enriches GFP + cells (CSC-like) in MDA-MB-231-SORE cell cultures. **A** Exposure schedule to 1–10 nM of doxorubicin (Dox) for 4 months. **B** FACS analysis demonstrates the enrichment of GFP + cells in cultures exposed to Dox; values are expressed as mean ± standard deviation (SD). Student’s test **p < 0.01 vs. control (n = 12)

To establish a resistant TNBC cell line, MDA-MB-231-SORE cells were cultured with (Dox) or without doxorubicin (control) under identical conditions and for the same period to allow for an appropriate comparison between the 2 conditions (Fig. 1A). Following treatment with increasing doxorubicin concentrations, the cells became enriched in GFP + populations (CSC-like) compared with control cells (Fig. 1B). We observed enrichment of GFP + cells from the first month of doxorubicin exposure; this enrichment was also seen in Hs578t cells (Supplementary Fig. 1).

### Enriched cells develop resistance to doxorubicin and increase their mammosphere-forming efficiency (MFE)

**Fig. 2:**
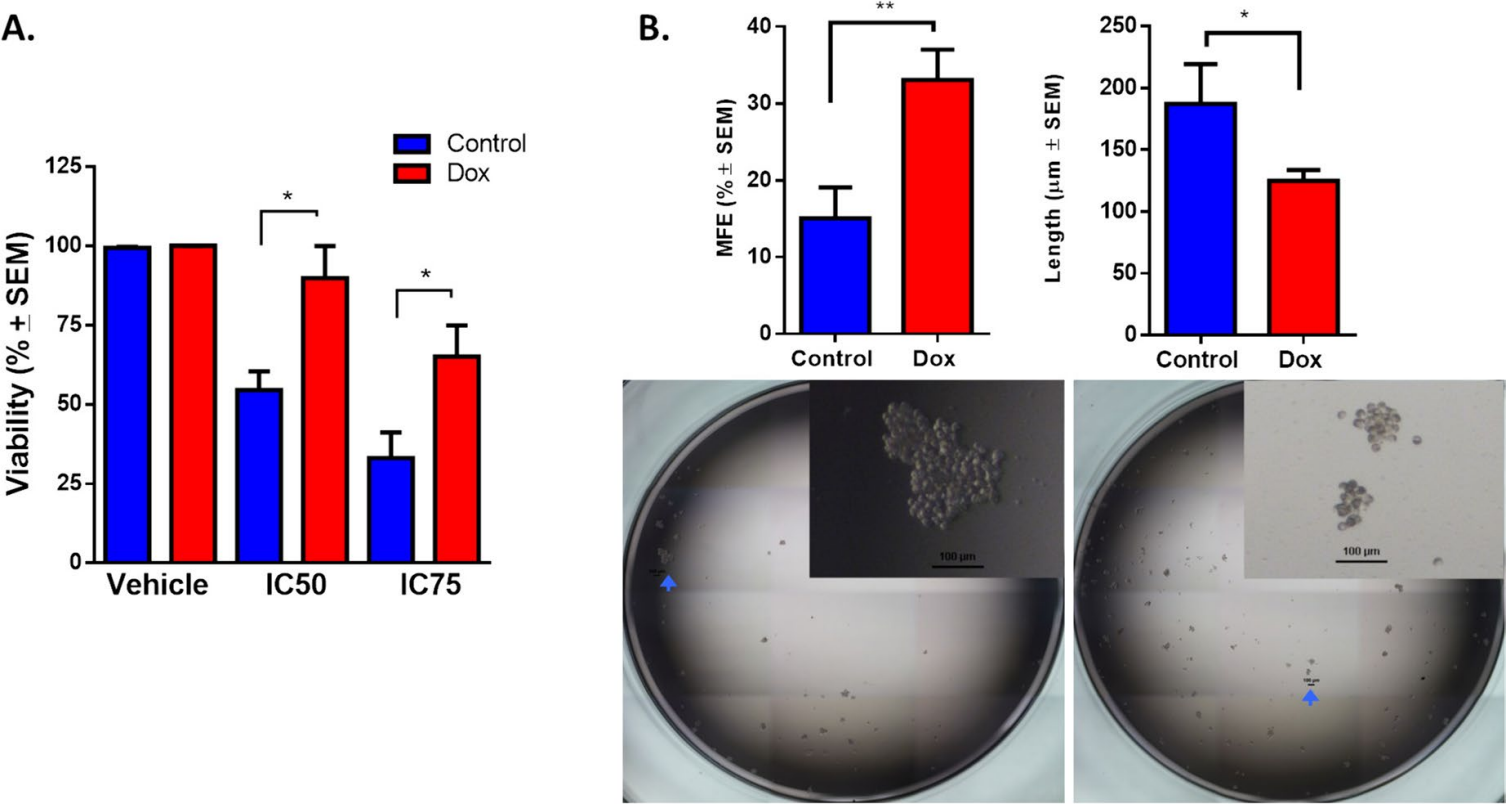
Continuous exposure to doxorubicin induces a drug-resistant phenotype and CSC-like characteristics in TNBC cells. **A** Cytotoxicity was compared between parental MDA-MB-231 cells (control) and MDA-MB-231 cells exposed to doxorubicin (Dox) at the IC50 or IC75 of Dox, evaluated by MTT assay. **B** Mammosphere formation in MDA-MB-231 cells exposed to doxorubicin for 4 months. Graphs show results from 4 assays evaluated in quadruplicate and the mean ± SEM. Representative pictures are shown; bar = 100 μm. Statistical significance was determined by Tukey’s multiple comparisons test against the control or student’s test; p < 0.05 (*), < 0.01 (**). MFE: mammospheres-forming efficiency

To evaluate resistance in cells that are continuously exposed to increasing concentrations of doxorubicin (1–10 nM), we analyzed the viability of cell culture at IC50 (0.43 μM) or IC75 (1.44 μM) for 72 h (Fig. 2A). The control cells showed 50% cell viability at 72 h, whereas Dox cells showed 90% and 70% viability at 0.43 μM and 1.44 μM doxorubicin, respectively. We confirmed that cells that were exposed to doxorubicin developed resistance, observing viability at concentrations 25-fold greater than the plasma concentrations of doxorubicin in patients (~ 18 nM) [27]. Given that CSCs have been reported as being intrinsically resistant to chemotherapy [22], we analyzed the viability of GFP + (CSC-like) and GFP- populations (non-CSCs) that were exposed to doxorubicin at IC50 in näive cultures. In these cultures, we noted a pronounced cytotoxic effect at the IC50 of doxorubicin, particularly affecting the GFP + populations (Supplementary Fig. 2). Gargini and colleagues also observed that doxorubicin induces cell death and cell cycle arrest in the CD44^high^/CD24^low^ population of MDA-MB-231 cells, demonstrating the susceptibility of CSC-likes [28]. The use of the reporter system (SORE-GFP) allowed us to compare the sensitivity of CSC and non-CSC. Thus, our results confirm that prolonged exposure to a low dose of doxorubicin is necessary to induce the drug-resistant phenotype, as other groups have reported [25, 26, 29, 30].

Next, we performed mammosphere assay to characterize the functional phenotype of doxorubicin-resistant cells. The mammosphere-forming efficiency (MFE) of Dox cells was twice that of control cells. The length of the spheres that were formed by Dox cells was lower than that of control cells (Fig. 2B). Our data show that enrichment of the GFP + population (CSC-like) acquires resistance and MFE due to continuous exposure to low concentrations of doxorubicin.

### Doxorubicin induces stemness and plasticity phenotype in GFP- populations

**Fig. 3:**
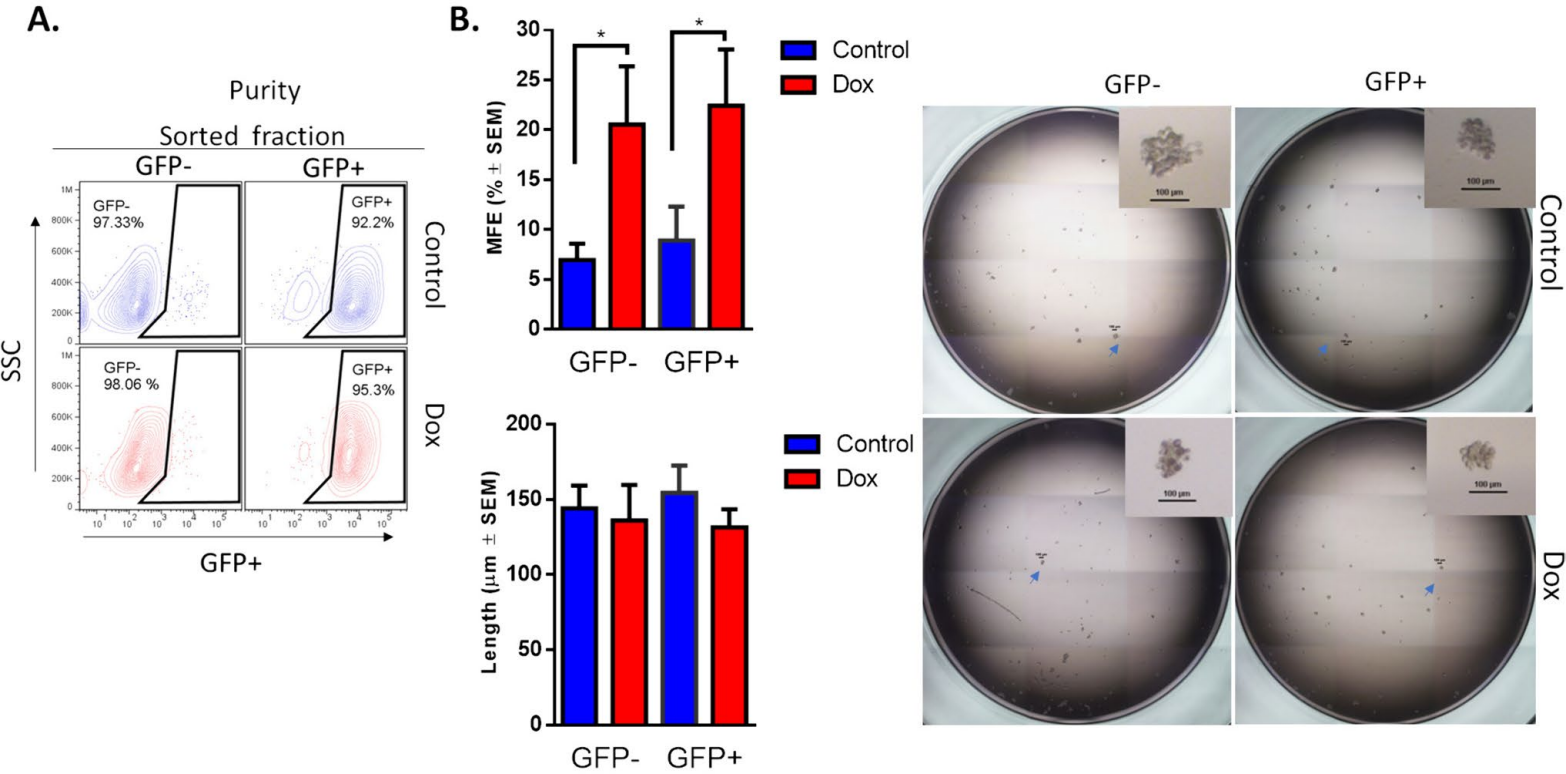
Doxorubicin increases mammosphere-forming efficiency in GFP + and GFP- cell populations. **A** FACS analysis of the purification efficiency of GFP + and GFP- populations. **B** Mammosphere formation in purified cells exposed to doxorubicin for 4 months. Graphs show results of 4 assays evaluated in quadruplicate and the mean ± SEM. Representative pictures are shown; bar = 100 μm. Statistical significance was determined by Tukey’s multiple comparisons test against the control; p < 0.05 (*). MFE: mammospheres-forming efficiency

To test whether GFP + cells (CSCs-like) that expand while acquiring doxorubicin resistance have a different stemness phenotype than GFP- cells (non-CSCs), we purified GFP + and GFP- cells (Fig. 3A) from Dox-resistant cultures and performed mammosphere assay (Fig. 3B). GFP + and GFP- cells from Dox-resistant cultures had a similar but higher MFE than purified cells from the control culture. However, the length of mammospheres was similar between the Dox-resistant and control cultures. We and others have reported that continuous stimulation with doxorubicin at a low dose increases the MFE in TNBC cells [25, 26]; however, in the current study, we found that both GFP + and GFP- populations from a culture that has been exposed to doxorubicin have a similar stemness phenotype, resulting in an increased MFE.

**Fig. 4:**
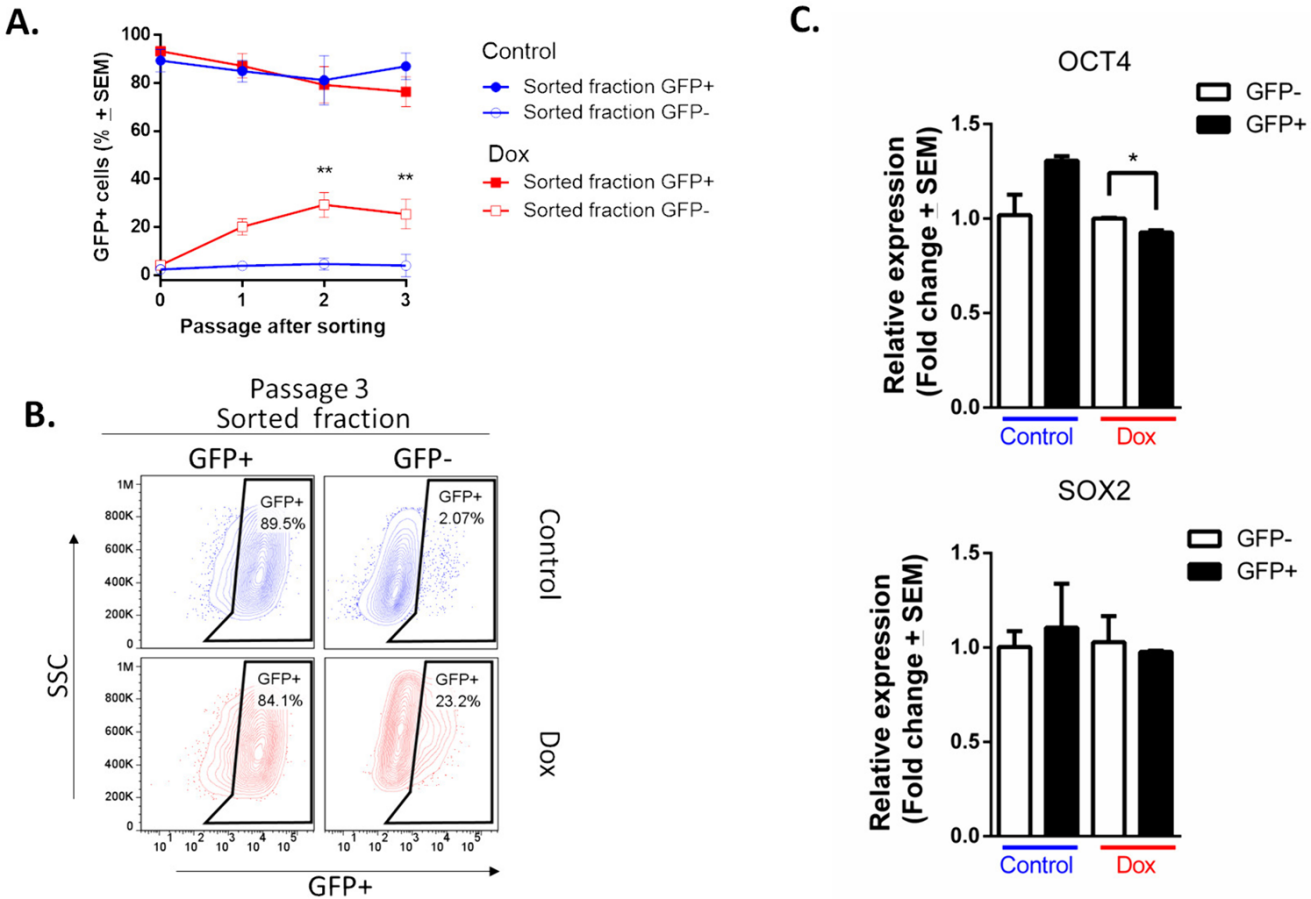
Plasticity of GFP- population (non-CSCs) from culture exposed to doxorubicin. **A** Time course showing that sorted GFP- populations have plastic potential to generate GFP + populations in culture. Three assays evaluated by duplicate and the mean ± SEM. Statistical significance was determined by Sidak’s multiple comparisons test against the control; p < 0.01 (**); **B** FACS analyses of third passage after sorting of GFP- population. **C**. Expression of SOX2 and OCT4 in purified cells exposed to doxorubicin for 4 months was measured by RT-qPCR. Error bars represent the standard error of the mean (SEM) of three biological replicates. Statistical significance was determined by student’s test; p = 0.035 (*)

It is widely accepted that CSCs can self-renew and give rise to more committed daughter cells, whereas regeneration of CSCs from more differentiated daughter cells is an infrequent phenomenon [31]. To analyze this principle in our enriched, resistant cells with a stemness phenotype, we culture purified cells (GFP + and GFP-) and determined the percentage of GFP + cells (Fig. 4). The GFP + populations regenerated GFP- cells, which increased the percentage slightly with passage in culture. In contrast, GFP- populations from the culture control were largely incapable of regenerating GFP + cells; notably, GFP- populations from the resistant culture rapidly regenerated GFP + cells, which increased the percentage with passage in culture (Fig. 4A). In Passage 3, the purification of GFP + populations resulted in a reduction in the GFP + percentage (89.5% and 84.1% in control and Dox, respectively). This decrease indicates the emergence of a GFP- populations. Conversely, the purification of GFP- populations from the resistant culture effected an increase in the GFP + percentage (23.2%), versus the 2.07% of GFP + cells in the GFP- populations that were purified from the control (Fig. 4B).

The expression of SOX2 and OCT4 was measured by RT-qPCR in GFP + and GFP- populations (Fig. 4C). In cells exposed to Dox, the relative expression of OCT4 is significantly higher in GFP- cells than in GFP + cells. For SOX2, no significant Dox-induced changes were identified. As expected, in control cells, the expression of both SOX2 and OCT4 is higher in the GFP + population than in the GFP − population. This result provides evidence for a positive regulatory role of OCT4 in Dox-induced resurgence in GFP + cells.

**Fig. 5:**
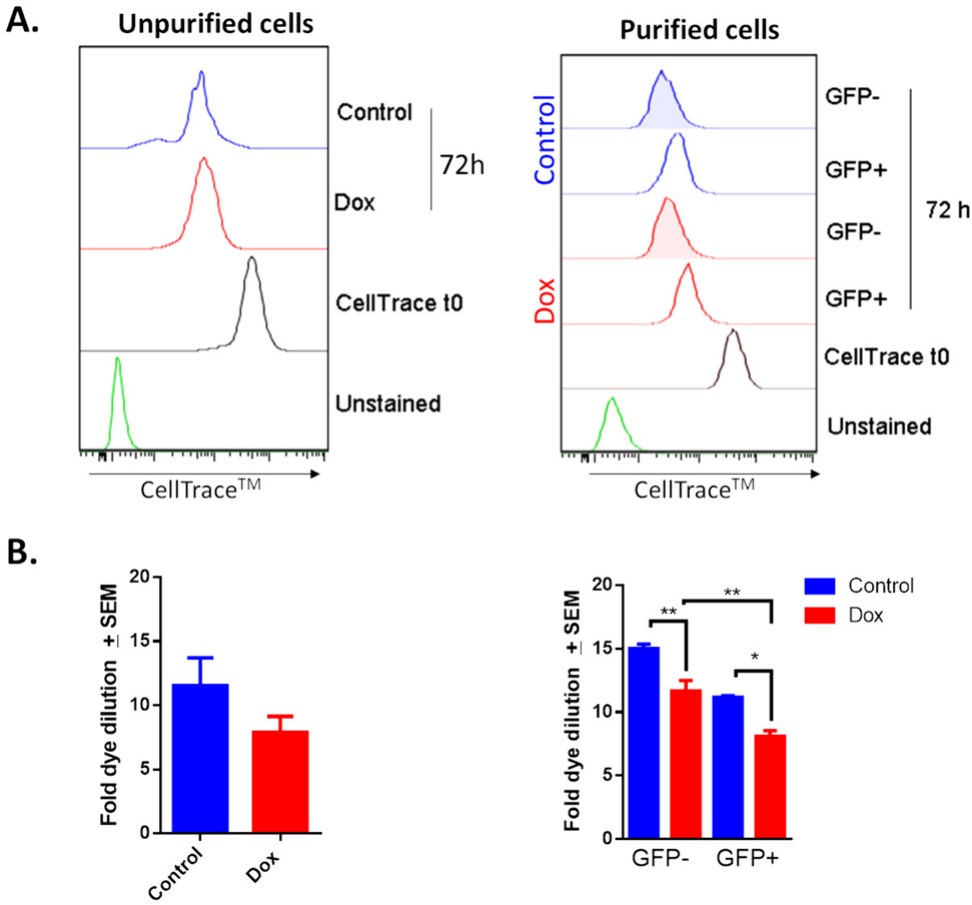
Exposure to doxorubicin increases cell proliferation in GFP- populations (plastic cells), compared with GFP + cells (CSC-like). **A**. FACS analysis demonstrating that doxorubicin (Dox) reduces cell proliferation in unsorted and sorted cell populations. In comparing GFP + and GFP- populations, the latter exhibits a higher proliferation rate. **B**. The graphs show data from two assays, each evaluated in triplicate and display the mean ± SEM. Statistical significance was determined using either the student´s test or Tukey’s multiple comparisons test against the control group; p < 0.05 (*); < 0.01 (**)

To determine whether the increase in GFP + percentage in GFP- populations that were purified from the resistant culture resulted from enhanced proliferative capacity due to doxorubicin, we analyzed cell proliferation. After sorting, purified cells were stained with CellTrace (t0), cultured for 72 h, and analyzed by flow cytometry (Fig. 5A). Purified and unpurified cells from the resistant culture showed lower proliferative capacity compared with control cells (Fig. 5 B). As has been reported, GFP- cells (non-CSCs) can proliferate faster than GFP + cells (CSCs) [32, 33].

### mRNA expression profiles of subpopulations purified from resistant culture

Phenotypic plasticity represents a paradigm for understanding cancer initiation, progression, and drug resistance that involves a broad set of phenomena, including the loss of lineage commitment and the acquisition of a stemness phenotype (dedifferentiation). Drug resistance is achieved by promoting the acquisition of a dedifferentiated state by increasing the expression of certain genes [34].

**Fig. 6:**
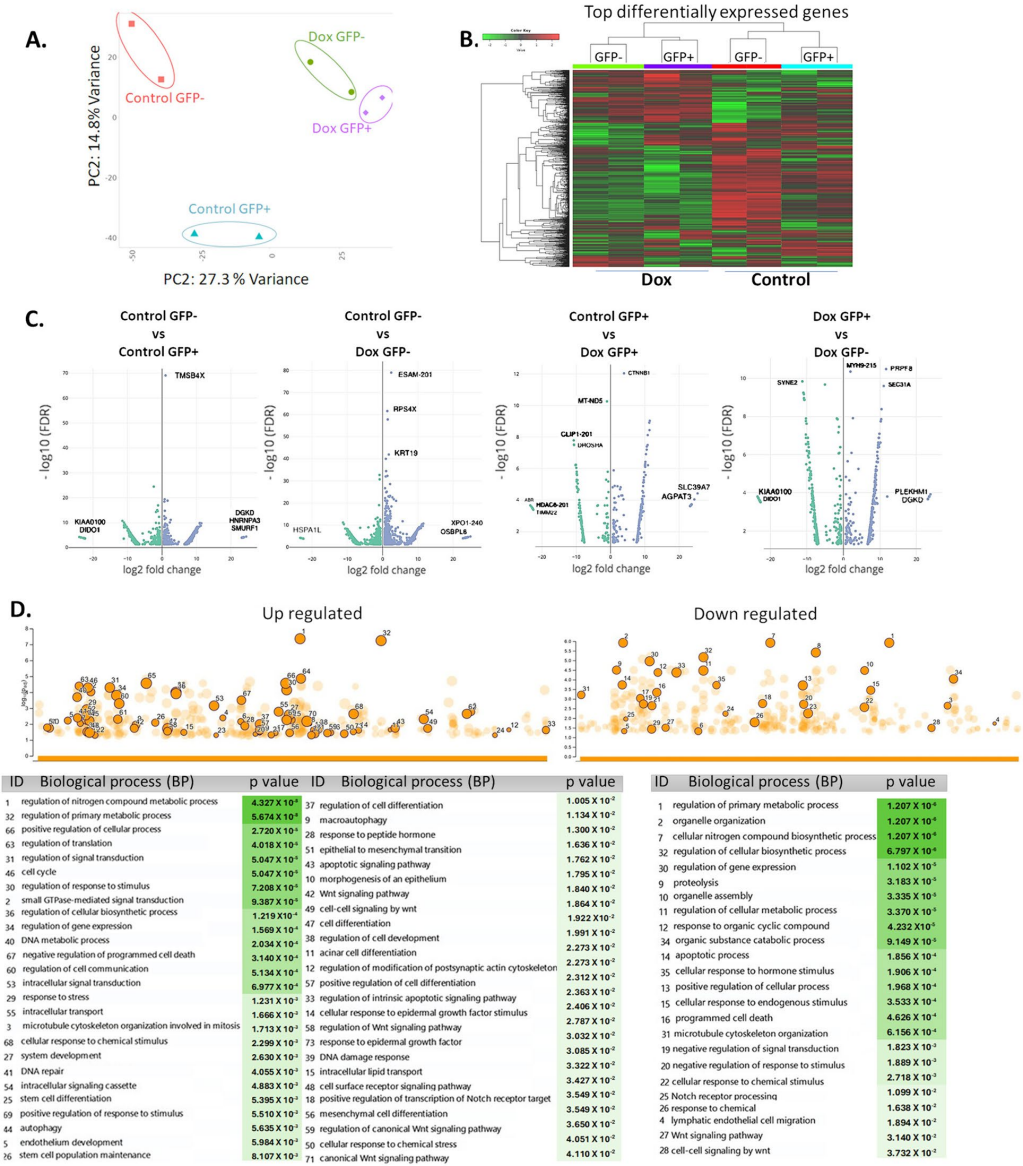

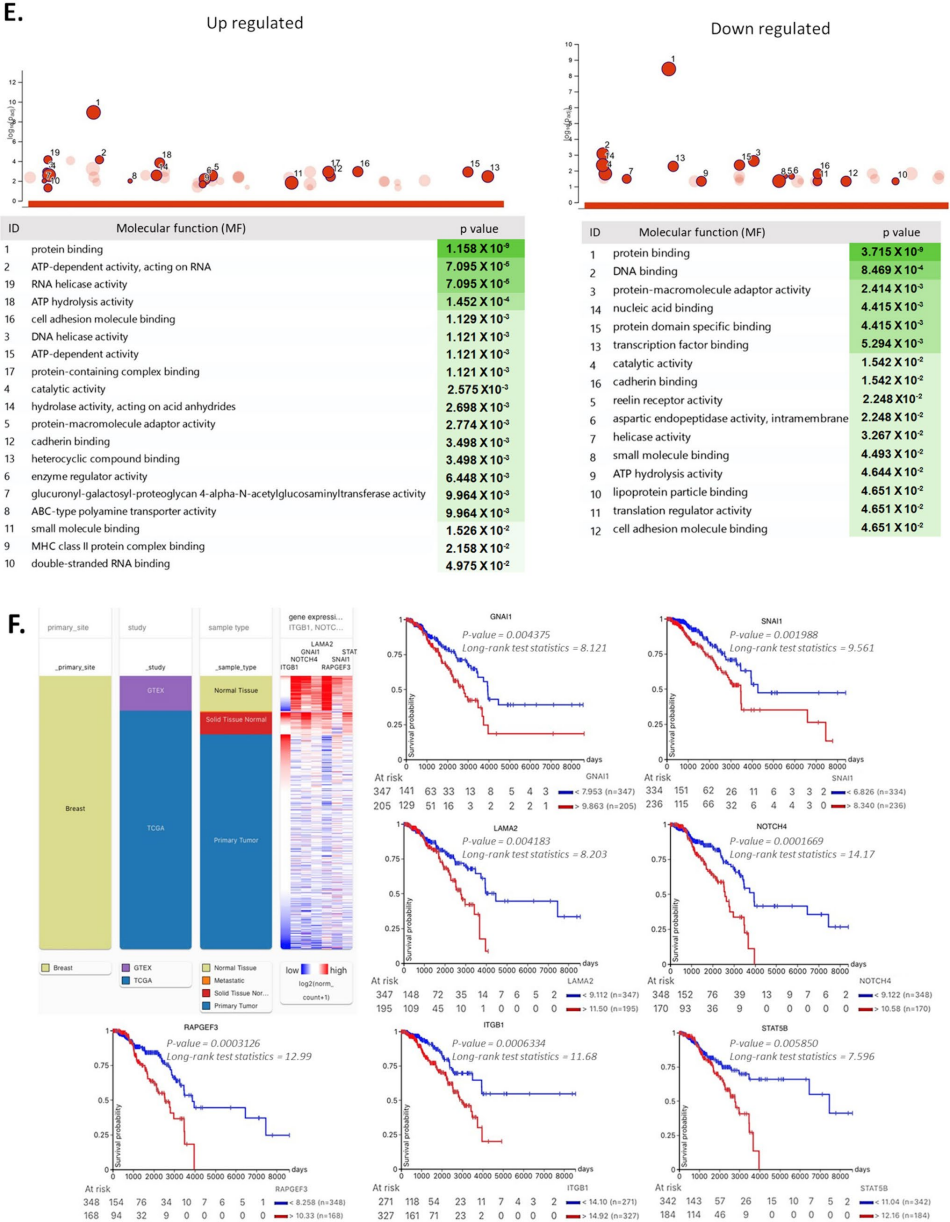
RNA-seq analysis of GFP + and GFP- populations exposed to doxorubicin. **A**. Principal component analysis (PCA) and subsequent visualization of PC1 and PC2 resulting in sample clustering. **B**. Heatmap of differentially expressed genes that are upregulated (red) or downregulated (green) in GFP + or GFP- populations (Control and Dox-resistant cells). **C**. Volcano plot of differential gene expression of GFP-/GFP + cells between control and doxorubicin-exposed conditions; genes acquired by DESeq2 (*p* value (–log10FDR) are plotted against fold-change (|log2-fold change|). Gene ontology analysis of differentially expressed genes in GFP- populations from the control culture and GFP- populations exposed to prolonged doxorubicin. **D**. Biological processes and **E**. molecular functions with adjusted *P* values less than 0.05, obtained from gProfiler. **F**. Analysis of *ITGB1, LAMA2, GNAI1, NOTCH4, RAPGEF3, STAT5B,* and *SNAl1* expression in breast tumors and clinical correlation. Heatmaps of mRNA levels of differentially expressed genes in breast tumors and their corresponding normal tissues. Comparison of expression of genes in breast tumors vs normal tissue (cohort: TCGA TARGET GTEx database of breast cancer patients obtained from UCSC Xena). Statistical analysis was performed using Welch’s t-test. N.S. non-significant. Overall survival curve in TNBC patients from TGCA cohort. All plots were generated using the UCSC Xena browser. Kaplan-Meyer analysis comparing patients with high (red) and low (blue) expression of genes; P values calculated by log-rank test. Threshold of Cox *p* value

RNA sequencing analysis (RNA-seq) was performed to analyze alterations in genes in the GFP- and GFP + populations from resistant and control cultures (Fig. 6). Principal component analysis (PCA) and subsequent visualization of PC1 and PC2 resulted in the clustering of samples by condition (Fig. 6A). A total of ~ 1000 genes were detected as differentially expressed and heuristically clustered into 4 groups, based on their expression profiles (Fig. 6B). The results from DESeq2, visualized in volcano plots, revealed 150–450 differentially expressed genes, based on predefined thresholds (adjusted p-value < 0.05 and |log2-fold change|), according to the combinations between pairs of doxorubicin and control from GFP + and GFP- populations (Fig. 6C). In the gene ontology analysis of upregulated genes (from control and Dox-resistant GFP- cells), several biological processes were identified, related to cellular response to chemical stimulus, mesenchymal cell differentiation, stem cell differentiation, stem cell population maintenance, and mechanism of DNA repair. Further, positive regulation of wnt signaling pathways and transcription of notch receptor targets was observed. Several identified processes were involved in self-renewal and differentiation of CSCs in several tissues and breast cancer [35]. The main downregulated genes were related to regulation of apoptosis and cellular metabolism (Fig. 6D). The molecular functions (Fig. 6E) were related to ABC-type transporter activity (upregulated genes) and protein-DNA binding and enzymatic activity (downregulated genes). The gene ontology for several distinct clusters of genes is provided in Supplementary Information (Supplementary Fig. 3). We identified 7 genes (ITGB1, SNAI1, NOTCH4, STAT5B, RAPGEF3, LAMA2, and GNAI1) that have been implicated in both stemness and the drug response and validated their relevance through an analysis of data from breast cancer patients in the TCGA database using UCSC Xena (Fig. 6F). Based on our RNA-seq analysis, treatment at low concentrations of doxorubicin induced differential gene expression in GFP- populations, explaining in part the presence of cells with a plastic phenotype, leading to enrichment of resistant cells with a stemness phenotype.

## Discussion

Doxorubicin (Dox) is widely used in the treatment of various types of cancer, including TNBC [30, 31]. However, although initial treatment with Dox is effective in controlling tumor growth, many patients relapse over time [36–38]. That is, the induction of drug resistance during chemotherapy is a main obstacle to successful cancer treatment.

In this study, we used a reporter system (SORE6) that allowed us to observe the enrichment of CSC-like (GFP +) populations in MDA-MB-231 and Hs578t cell lines following prolonged and continuous exposure to low doxorubicin concentrations. After 4 months of exposure to doxorubicin, MDA-MD-231 cells exhibited a drug-resistant phenotype and acquired CSC properties, leading to increased mammosphere formation efficiency. This finding is consistent with several groups that have reported the enrichment of CSCs and drug resistance in TNBC cells following exposure to doxorubicin, using treatment regimens with concentrations from 5 nM-250 nM from 1 to 24 months of exposure [25, 26, 29, 39]. Several studies have determined the effect of doxorubicin on CSCs using markers such as CD44 + /CD24- and ALDH + . In clinical studies whit human breast cancer tissue obtained before and after chemotherapy found that CD44 + /CD24- or ALDH1 + tumor cells significantly increased after chemotherapy [20, 40]. In vitro studies shown that the expression of CD44 and OCT3/4 increases with the acquisition of doxorubicin resistance in MDA-MB-231 cells [25]. This suggests that regardless of the CSC study strategy (reporter system, immunohistochemistry, or western blot), treatment with doxorubicin leads to the enrichment of CSCs.

Evidence suggests that the treatment of cancer cells with therapeutics constitutes a novel source of cell plasticity and stemness phenotype within tumors. Chemotherapy can promote or enhance stem cell-related phenotypes in tumor cells [41, 42]. In our study, the reporter system allowed us to purify non-CSCs (GFP- population) and CSCs (GFP + population) and determine that continuous exposure to doxorubicin increases the mammosphere-forming efficiency in both populations, suggesting that such treatment induces stem cell-like properties in differentiated cancer cells, leading to the outgrowth of previously differentiated.

The genes and biological processes that were identified in the RNA-seq analysis of non-CSCs (GFP- population) that were exposed to Dox suggest that GFP- cells are capable of inducing stem cell differentiation and maintaining the CSC population (GFP + population). The transcriptome findings were confirmed, because we observed that CSCs (GFP + population) increase with each passage. As expected, control cells did not give rise to CSCs. In contrast, non-CSCs from the control culture were incapable of regenerating a CSC population. Our findings suggest that GFP- cells (non-CSCs) that were derived from resistant cultures of TNBC cells develop a plastic cell phenotype, allowing them to acquire the hallmark characteristics and phenotypes of CSCs. We observed that the GFP-negative populations that were derived from these drug-resistant cultures quickly reestablished GFP + populations. This resurgence in GFP + cells could be attributed to the upregulation of OCT4 in response to exposure to doxorubicin, rather than a change in the proliferative capacity of the GFP + population. Therefore, demonstrating that OCT4 plays a relevant role in plasticity. It has been reported that expression of OCT4 is sufficient to reprogram a cell based on a signal-response[43]. Cheng and colleagues reported that the expression levels of Oct-4 and c-Myc increase in doxorubicin-resistant TNBC cells, while the expression of Sox2 does not change significantly [26], which agrees with our findings.

Notably, we found that CSCs that were exposed to Dox at IC50 were more susceptible to apoptosis than non-CSCs, suggesting that only a small population has intrinsic resistance to Dox and that these population are responsible for recurrence, as proposed by several groups [10, 40, 41]. Our results show that cells that are exposed to low concentrations of Dox regulate genes that are involved in apoptosis, the response to chemical stimulus, and metabolic activity. Thus, we conclude that high concentrations of Dox exert selective pressure on heterogeneous tumor cells, leading to the outgrowth of surviving resistant cells and inducing the death of sensitive cells, whereas low concentrations effect the conversion of non-CSCs to maintain the CSC population. These findings have clinical implications, because plasma concentrations in patients are approximately 18 nM [27]. Further, it is evident that heterogeneity exists even within CSCs and that they may respond differently to treatments in a concentration-dependent manner [22], which could lower the overall sensitivity to therapy over time or increase the relative proportion of cells in residual tumors with tumorigenic properties after treatment. Although repeated exposure to Dox leads to the development of drug-resistant cancer cells [25, 26, 29, 40, 44, 45], our understanding of the molecular mechanisms and pathways that are involved remains poor.

This study has clearly demonstrated that resistant cells acquire CSC properties but that the plasticity of cancer cells, including CSCs, is a complex process that can arise through dedifferentiation during EMT. RNAseq analysis allowed us to identify molecules that are involved in this phenotypic plasticity and the transition from a state of differentiated cells to one of stemness. For example, in breast cancer cells, ITGB1 was identified as participating in adhesion to various extracellular matrices, self-renewal, and chemoresistance [46]. The transcription factor SNAI1 mediates epithelial-mesenchymal transition; thus, loss of SNAI1 may lead to lineage plasticity, which could explain some of the phenotypes in heterogeneous breast cancers [47]. The Notch4 signaling pathway has been widely studied, and its dysregulation promotes resistance to therapies by enriching a small population of resistant cells [48, 49]. STAT5 has been identified as a key promoter in Dox resistance in breast cancer by positively regulating ABCB1 expression [50]; in our study, we also observed positive regulation of ABC transporters. Studies on LAMA2 [51] and RAPGEF3 [50] have identified their role in cancer progression, through the regulation of signaling pathways. GNAI1 transduces extracellular signals that function in several physiological processes, including proliferation, differentiation, and lipid metabolism, which are related to tumor growth by affecting gene expression and regulating the inflammatory response, epithelial-mesenchymal transition, and angiogenesis [52].

One of the limitations of this study is the lack of validation of the differential expression analysis results. However, it is worth mentioning that our results are promising because the changes in OCT4 expression identified by qPCR in GFP- cells are consistent with the biological processes identified through gene ontology. Nonetheless, further validation can reveal the importance of the genes identified following doxorubicin exposure in drug resistance and cell plasticity.

Understanding the molecular mechanisms of drug resistance and induction of stemness by therapeutic agents for cancer treatment has clinical significance in improving the efficacy of chemotherapy in TNBC patients and could identify new therapeutic targets.

## Supplementary Information

Below is the link to the electronic supplementary material.

Supplementary file1 (DOCX 1444 KB)

## Data Availability

Sequence data that support the findings of this study have been deposited in GEO Data Set in personalized upload space /uploads/314313797@quimica.unam.mx_UfGv6002.
