# Supplementary material for: Continuous exposure to doxorubicin induces stem cell-like characteristics and plasticity in MDA-MB-231 breast cancer cells identified with the SORE6 reporter
Source: Cancer Chemother Pharmacol. 2024 Aug 24;94(4):571–83. doi: 10.1007/s00280-024-04701-4 (PMC11438702; doi:10.1007/s00280-024-04701-4)
Supplement: Supplementary file 1 — Supplementary file1 (DOCX 1444 KB) [file 280_2024_4701_MOESM1_ESM.docx]

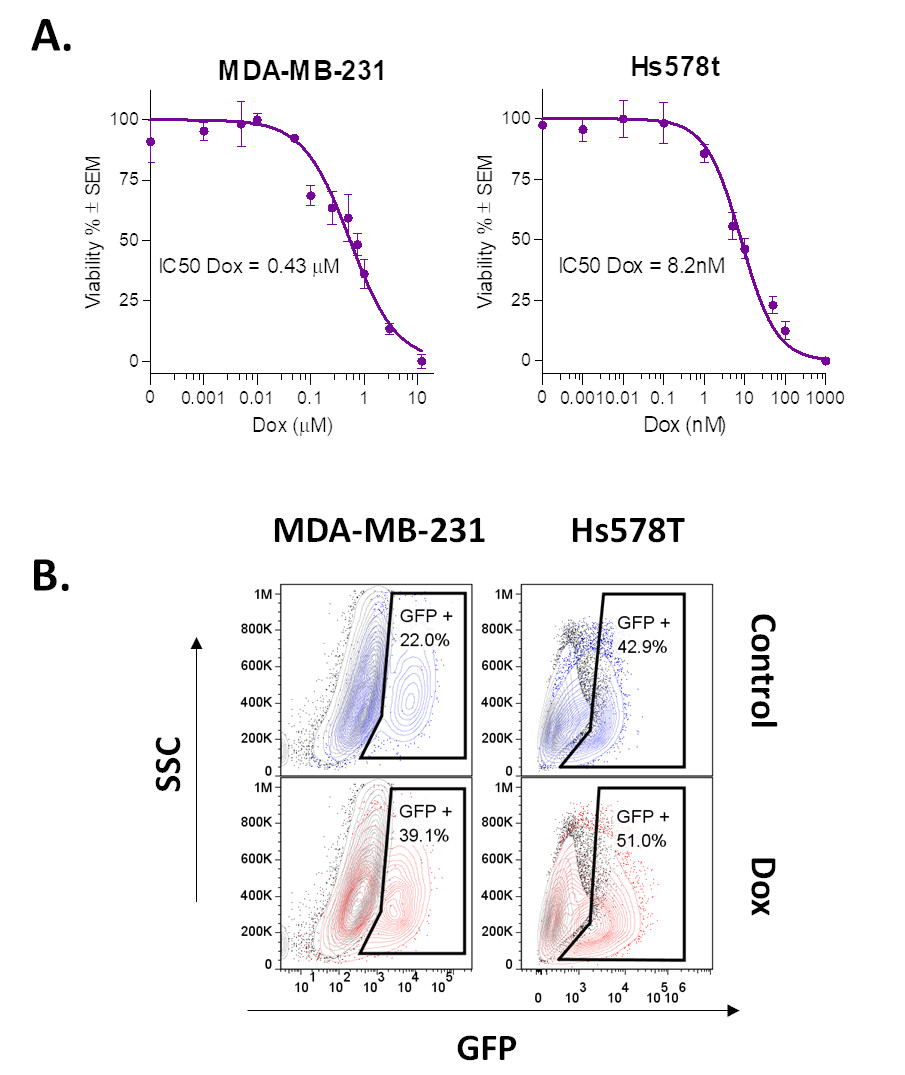


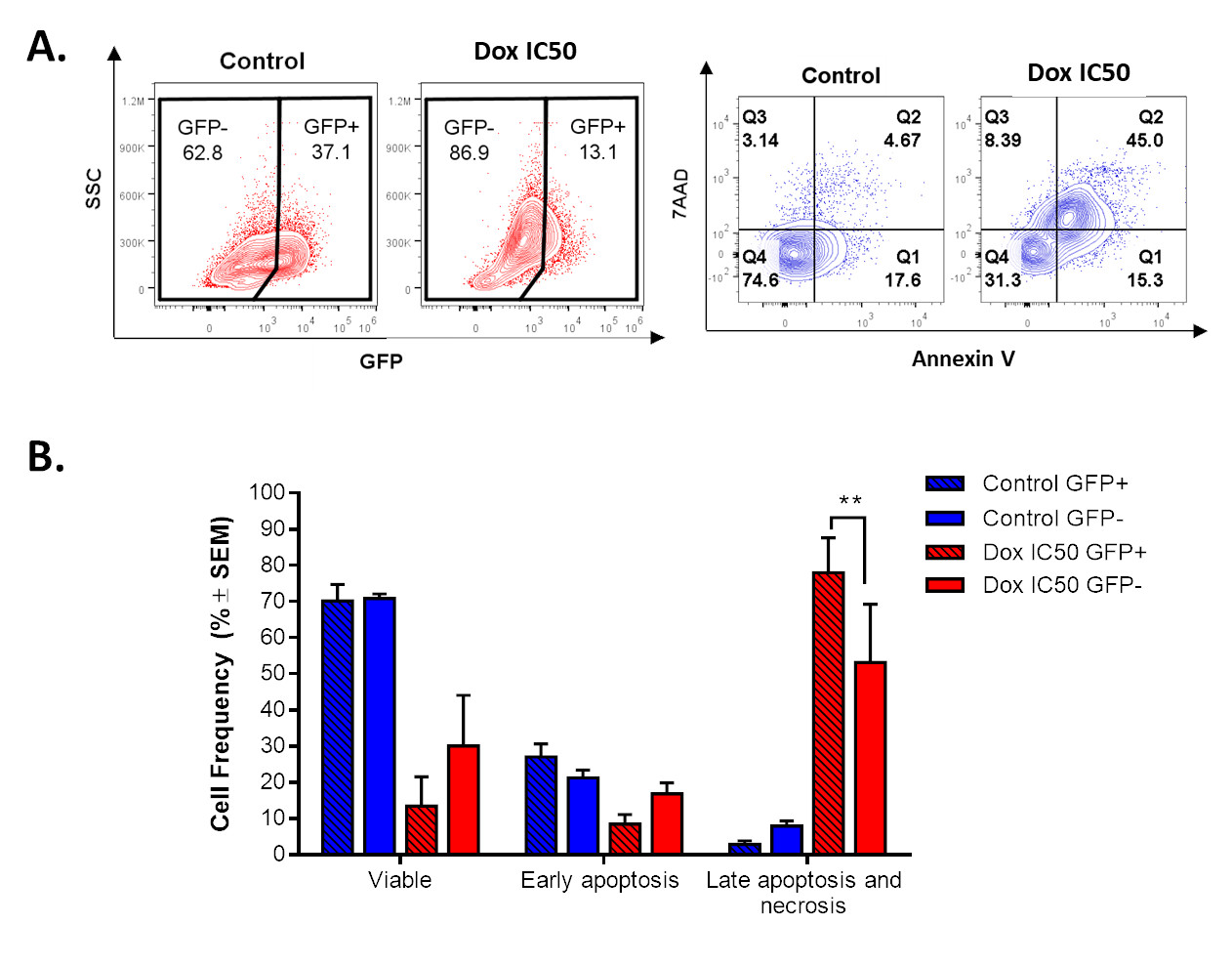


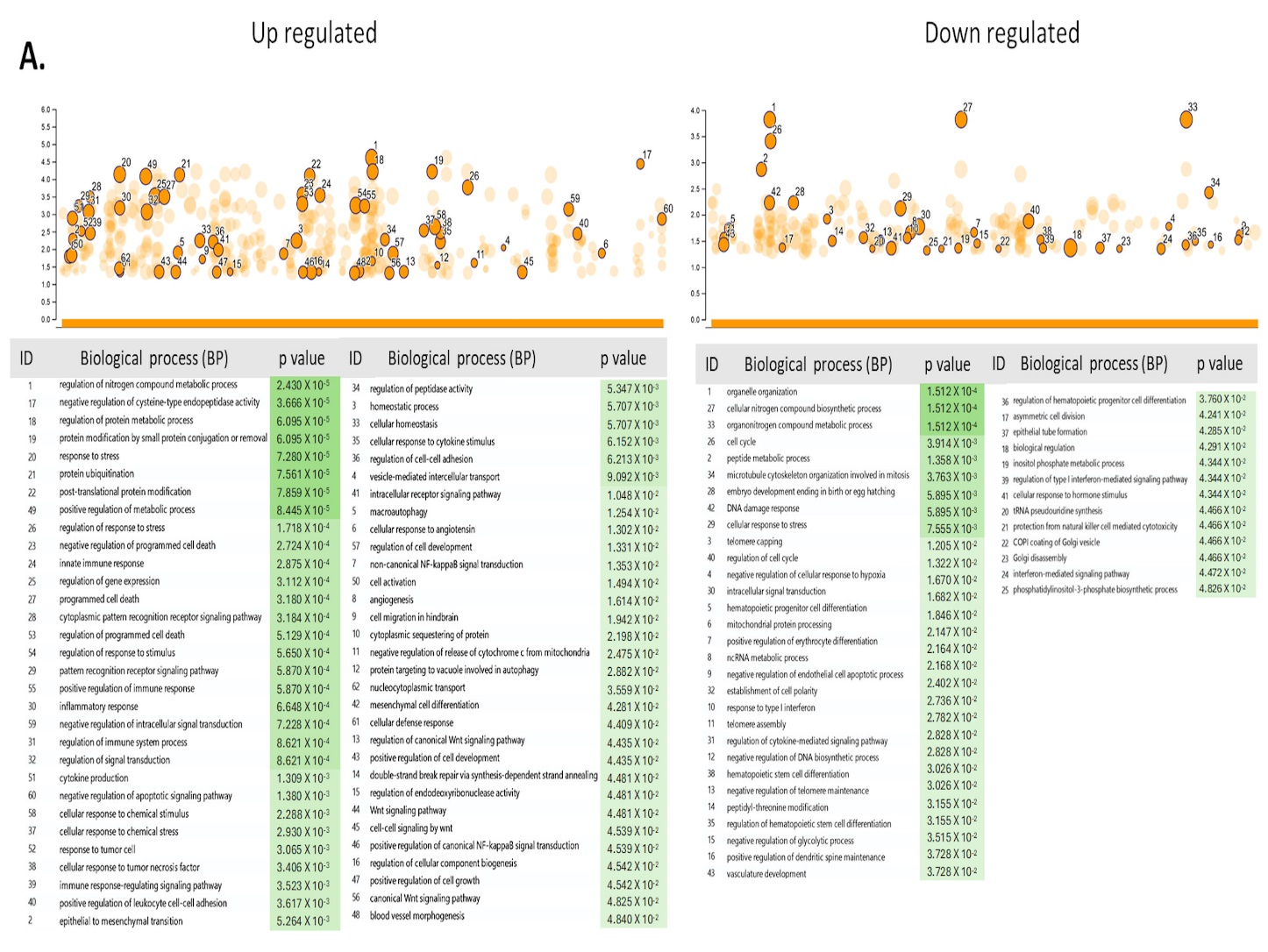


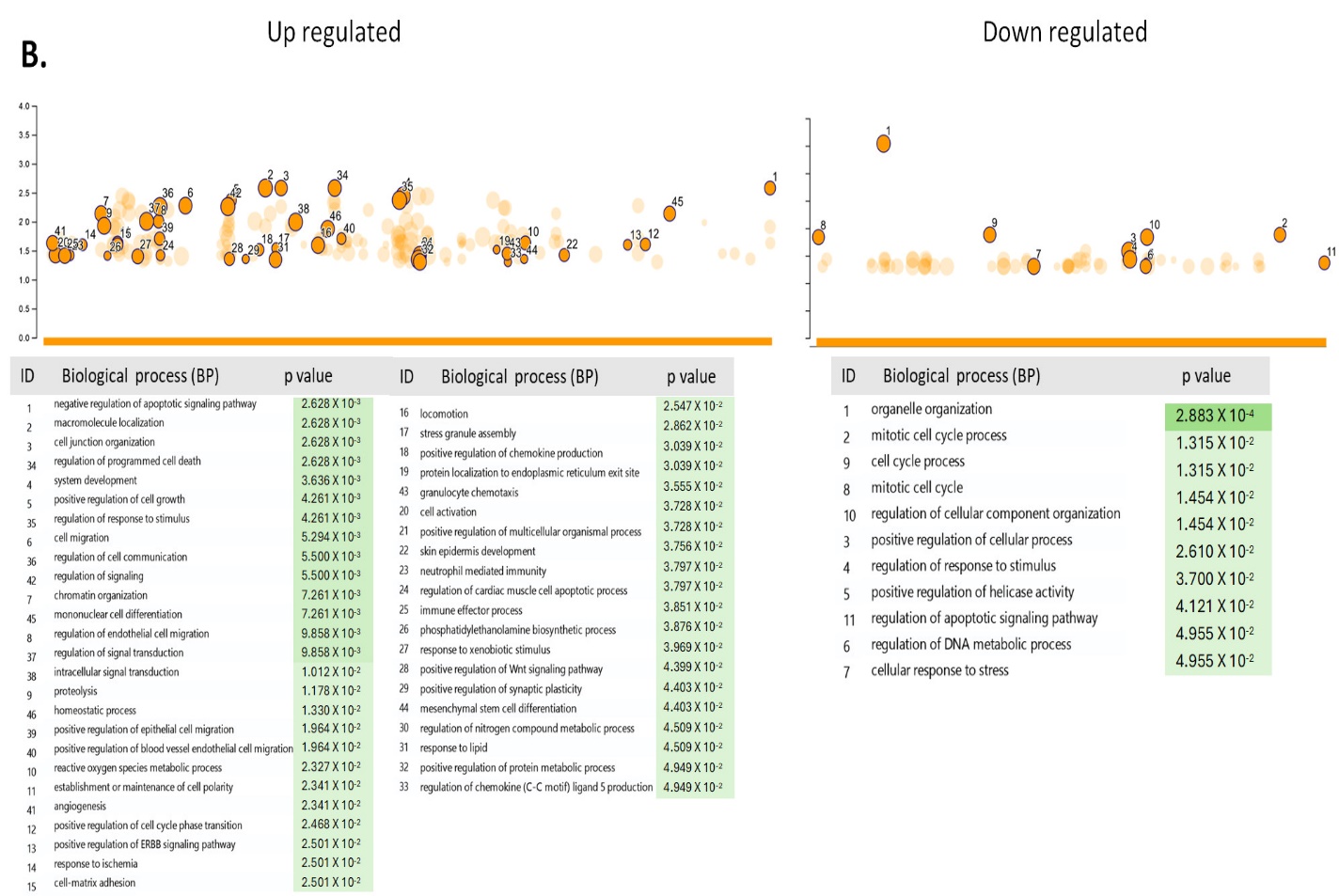


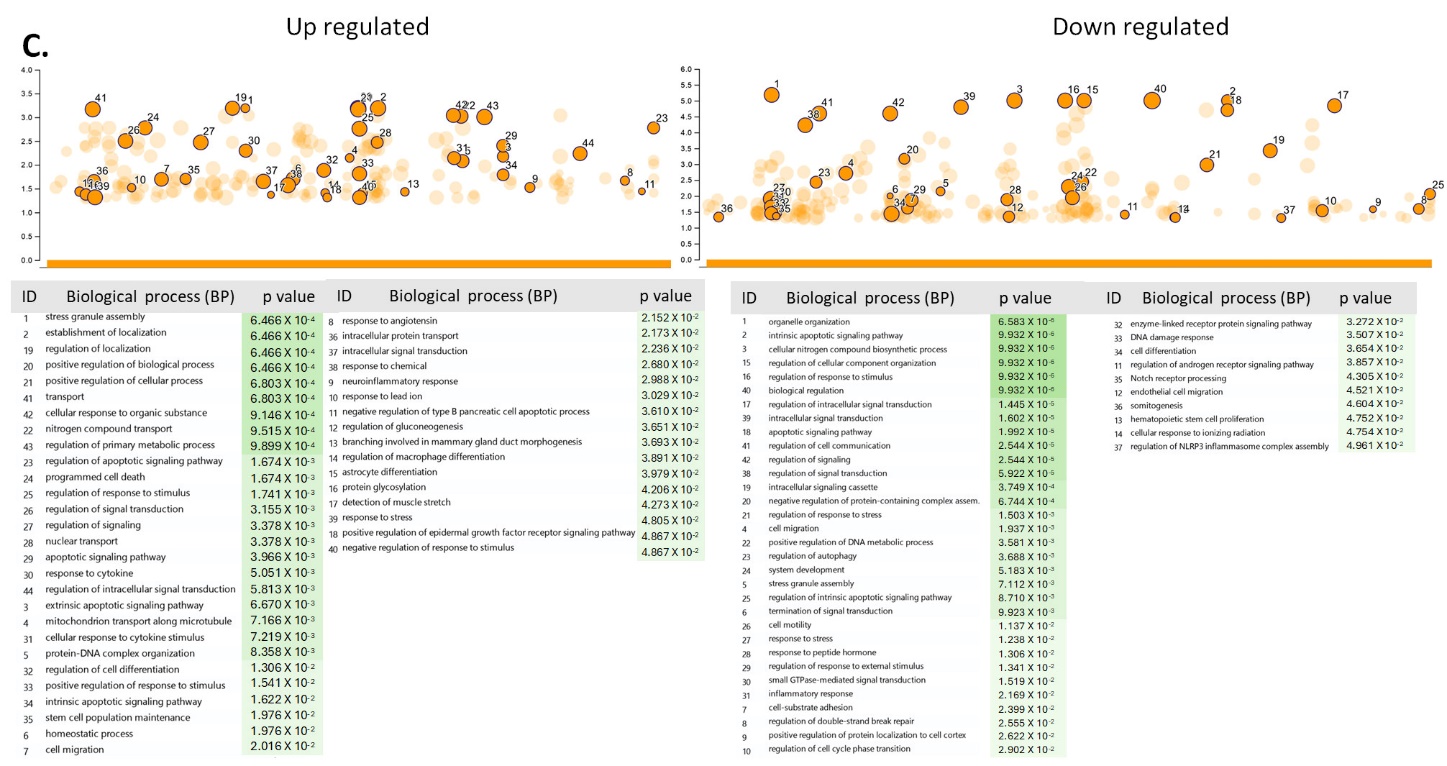


**Supplementary Information**

**Supplementary Figure 1. Establishment of conditions for long-term exposure at low concentrations of doxorubicin.** A. Based on the dose-dependent curve of doxorubicin (Dox), evaluated by MTT assay, we chose to expose MDA-MB-231 cells to subtoxic concentrations of 1-10 nM Dox and Hs578t cells to 0.1 nM Dox. Further, we determined the IC50 value for subsequent assays. B. FACS analysis, revealing the enrichment of GFP+ cells (CSCs) in MDA-MB-231 and Hs578t cultures following continuous exposure to 1 nM or 0.1 nM Dox over 1 month. The results are representative of 3 independent experiments. MTT, 3-(4,5-dimethyl-2-thiazolyl)-2,5-diphenyl-2-H-tetrazolium bromide; SEM, standard error media; FACS, fluorescence-activated cell sorting; GFP, green fluorescent protein.

**Supplementary Figure 2. Doxorubicin (Dox) induces apoptosis in GFP+ (CSC-like) and GFP- cells (non-CSCs).** A. FACS analysis shows that the IC50 of Dox reduces the GFP+ population and promotes apoptosis after 72 h of exposure. B. Dox increases the frequency of late apoptosis in GFP+ and GFP- populations, with a significant effect in the GFP+ population. SEM, standard error media; FACS, fluorescence-activated cell sorting; GFP, green fluorescent protein. Statistical significance was determined by Tukey’s multiple comparisons p < 0.01 (**).

**Supplementary Figure 3. Gene ontology analysis of differentially expressed genes in GFP+ and GFP- populations.** A**.** Biological processes associated with differential gene expression between GFP- and GFP+ populations from the control group. B. Biological processes associated with differential gene expression between GFP+ and GFP+ populations from the control and Dox groups. C. Biological processes associated with differential gene expression between GFP- and GFP+ populations from the Dox group. Data were adjusted P values less than 0.05, obtained from gProfiler, are shown.
